# The League of Mercy

**Published:** 1905-01-07

**Authors:** 


### THE LEAGUE OF MERCY.

Last week we dealt with the gratifying success of the League of Mercy, and the causes which underlie it. To-day we give the full figures for each district, corrected to December 31st, 1904.

LIST OF CONTRIBUTIONS, 1903-1904.

The following table shows the results and progress of the various divisions during 1903 and 1904:—

| District | Amount Received | | Increase | Decrease |
|---|---|---|---|---|
| | 1904 | 1903 | 1904 | 1903 |
| | £ | £ | £ | £ |
| Kensington (South) ... | 1,316 | 946 | 370 | — |
| Fulham ... ... ... | 1,062 | 1,049 | 13 | — |
| Berks ... ... ... | 778 | 16 | 762 | — |
| St. George, Hanover Sq.... | 749 | 712 | 37 | — |
| Paddington (South) ... | 706 | 428 | 278 | — |
| Sevenoaks ... ... | 622 | 547 | 75 | — |
| St. Pancras (North) ... | 534 | 383 | 151 | — |
| Epsom—Esher ... ... | 513 | 364 | 149 | — |
| Kingston—Surbiton ... | 498 | 251 | 247 | — |
| Hampstead ... ... | 471 | 363 | 108 | — |
| Chelsea ... ... ... | 460 | 159 | 301 | — |
| Westminster ... ... | 444 | 545 | — | 101 |
| Tonbridge ... ... | 426 | 420 | 6 | — |
| Marylebone (West) ... | 389 | 382 | 7 | — |
| Tower Hamlets (Whitechapel)... ... | 341 | 344 | — | 3 |
| Paddington (North) ... | 351 | 552 | — | 201 |
| Strand ... ... ... | 341 | 21 | 320 | — |
| Lambeth (Norwood) ... | 335 | 266 | 69 | — |
| Marylebone (East) ... | 323 | 170 | 153 | — |
| Deptford—Brockley ... | 315 | 265 | 50 | — |
| Chelmsford ... ... | 292 | 1 | 291 | — |
| Ealing ... ... ... | 283 | 387 | — | 104 |
| Lewisham ... ... | 257 | 180 | 77 | — |
| Guildford ... ... | 247 | 641 | — | 394 |
| Woolwich ... ... | 224 | 243 | — | 19 |
| City of London ... ... | 203 | 221 | — | 18 |
| Hertford ... ... ... | 195 | 180 | 15 | — |
| Epping ... ... ... | 187 | 132 | 55 | — |
| Eastbourne ... ... | 181 | 202 | — | 21 |
| Hammersmith ... ... | 183 | 160 | 23 | — |
| Kensington (North) ... | 170 | 58 | 112 | — |
| Watford ... ... ... | 161 | 7 | 154 | — |
| Maldon ... ... ... | 161 | 138 | 23 | — |
| Tower Hamlets (Mile End) | 156 | 112 | 44 | — |
| Romford ... ... ... | 145 | 129 | 16 | — |
| Chertsey ... ... ... | 143 | 182 | — | 39 |
| Richmond ... ... | 142 | 339 | — | 197 |
| Greenwich ... ... | 131 | 87 | 44 | — |
| Brentford... ... ... | 127 | 100 | 27 | — |
| St. Pancras (West) ... | 123 | 31 | 92 | — |
| Hackney (North) ... | 110 | 46 | 64 | — |
| Isle of Thanet ... ... | 104 | 2 | 102 | — |
| Wimbledon ... ... | 103 | 108 | — | 5 |
| Medway ... ... ... | 100 | 100 | — | — |
| Horsham ... ... ... | 91 | 85 | 6 | — |
| Islington (West) ... | 90 | 118 | — | 28 |
| Finsbury (East) ... ... | 90 | 140 | — | 50 |
| Croydon ... ... ... | 82 | 101 | — | 19 |
| Colchester ... ... | 81 | 55 | 26 | — |
| Enfield ... ... ... | 80 | 64 | 16 | — |
| Reigate ... ... ... | 73 | 72 | 1 | — |
| Hythe ... ... ... | 63 | 62 | 1 | — |
| Ashford ... ... ... | 57 | 77 | — | 20 |
| Walthamstow ... ... | 51 | 59 | — | 8 |
| Southwark (Bermondsey) | 47 | 33 | 14 | — |
| Lambeth (Brixton) ... | 45 | — | 45 | — |
| Faversham ... ... | 43 | 78 | — | 35 |
| Chichester ... ... | 42 | 21 | 21 | — |
| Harwich ... ... ... | 37 | 31 | 6 | — |
| Lewes ... ... ... | 35 | — | 35 | — |
| St. Albans ... ... | 35 | 31 | 4 | — |
| Dartford ... ... ... | 31 | 40 | — | 9 |
| Streatham ... ... | 30 | — | 30 | — |
| Battersea and Clapham... | 29 | 22 | 7 | — |
| Bethnal Green (N.E.) ... | 25 | 25 | — | — |
| St. Pancras (East) ... | 21 | 34 | — | 13 |
| Chatham ... ... ... | 18 | — | 18 | — |
| Southwark (West) ... | 16 | — | 16 | — |
| Harrow ... ... ... | 27 | 22 | 5 | — |
| Camberwell (Peckham)... | 11 | 23 | — | 12 |
| Hackney (Central) ... | 8 | 11 | — | 3 |
| Brighton ... ... ... | 8 | 25 | — | 17 |
| West Ham ... ... | 6 | — | 6 | — |
| Lambeth (North) ... | 5 | 6 | — | 1 |
| Southwark (Rotherhithe) | 5 | — | 5 | — |
| Bucks (South) ... ... | 4 | — | 4 | — |
| Islington (East) ... ... | 6 | 11 | — | 5 |
| Camberwell (Dulwich) ... | 2 | 4 | — | 2 |
| Worthing ... ... ... | 2 | — | 2 | — |
| Newington (West) ... | 1 | — | 1 | — |
| Rye ... ... ... | 1 | — | 1 | — |
| Finsbury (Holborn) ... | 1 | 18 | — | 17 |
| Wandsworth ... ... | — | 5 | — | 5 |
| Hitchin ... ... ... | — | 8 | — | 8 |
| Totals ... ... | 16,401 | 13,250 | 4,505 | 1,354 |

(Net increase 1904 over 1903—£3,151).

